# Shark Predation on Migrating Adult American Eels (*Anguilla rostrata*) in the Gulf of St. Lawrence

**DOI:** 10.1371/journal.pone.0046830

**Published:** 2012-10-17

**Authors:** Mélanie Béguer-Pon, José Benchetrit, Martin Castonguay, Kim Aarestrup, Steven E. Campana, Michael J. W. Stokesbury, Julian J. Dodson

**Affiliations:** 1 Université Laval, Département de Biologie, Pavillon Vachon, Québec, Québec, Canada; 2 Institut Maurice-Lamontagne , Fisheries and Oceans Canada, Mont-Joli, Québec, Canada; 3 Technical University of Denmark, National Institute of Aquatic Resource, Silkeborg, Denmark; 4 Canadian Shark Research Laboratory at the Bedford, Institute of Oceanography, Dartmouth, Nova Scotia, Canada; 5 Department of Biology, Acadia University, Wolfville, Nova Scotia, Canada; University of California Davis, United States of America

## Abstract

In an attempt to document the migratory pathways and the environmental conditions encountered by American eels during their oceanic migration to the Sargasso Sea, we tagged eight silver eels with miniature satellite pop-up tags during their migration from the St. Lawrence River in Québec, Canada. Surprisingly, of the seven tags that successfully transmitted archived data, six were ingested by warm-gutted predators, as observed by a sudden increase in water temperature. Gut temperatures were in the range of 20 to 25°C—too cold for marine mammals but within the range of endothermic fish. In order to identify the eel predators, we compared their vertical migratory behavior with those of satellite-tagged porbeagle shark and bluefin tuna, the only endothermic fishes occurring non-marginally in the Gulf of St. Lawrence. We accurately distinguished between tuna and shark by using the behavioral criteria generated by comparing the diving behavior of these two species with those of our unknown predators. Depth profile characteristics of most eel predators more closely resembled those of sharks than those of tuna. During the first days following tagging, all eels remained in surface waters and did not exhibit diel vertical migrations. Three eels were eaten at this time. Two eels exhibited inverse diel vertical migrations (at surface during the day) during several days prior to predation. Four eels were eaten during daytime, whereas the two night-predation events occurred at full moon. Although tagging itself may contribute to increasing the eel's susceptibility to predation, we discuss evidence suggesting that predation of silver-stage American eels by porbeagle sharks may represent a significant source of mortality inside the Gulf of St. Lawrence and raises the possibility that eels may represent a reliable, predictable food resource for porbeagle sharks.

## Introduction

The American eel (*Anguilla rostrata*) is a widely-distributed diadromous fish with a continental population ranging from southwestern Greenland to the north coast of Venezuela [1], [2], [3]. The facultative catadromous life cycles of the American eel and its congeneric North Atlantic species, the European eel (*A. anguilla*), have fascinated biologists for over a century. Since the pioneering work of Schmidt [4], [5], who identified the southwestern Sargasso Sea as the spawning area of both North Atlantic species (based on the capture of their distinctive leptocephalus larvae), the search has been on to home in on and characterize the specific spawning areas [6] as well as to characterize the migratory pathways of reproductive eels across vast expanses of open ocean (e.g. [7]). The interest in the reproductive ecology of the two species has taken on a degree of urgency since the early 1980's with the documentation of significant declines in the abundance and recruitment of both the American and European eel [8], [9] .

Various hypotheses with respect to the cause of this decline have been proposed, including changes in oceanographic conditions impacting the drift, survival and eventual recruitment of the juvenile stages to continental waters [10], [11]. However, little attention has been paid to how oceanographic conditions may affect the migration and survival of the adult migratory stage (known as the silver eel phase). Work on the European eel in Denmark revealed high mortality of silver eels in fjords where they reside for several months before initiating their oceanic migration, but fishing appeared to be the prime reason for the mortality [12], [13]. Nothing is known about marine mortality rates caused by oceanographic conditions, predation-related mortality, or the success rate of silver eels reaching the purported spawning grounds.

Recent research efforts using a new generation of miniature, archival, satellite tags (commonly referred to as pop-up tags) to quantify migratory pathways have proven successful in revealing marked diel vertical migrations (DVM) among anguillid species (longfin eel (*Anguilla dieffenbachii*), a Pacific anguillid [14]; Japanese eel (*Anguilla japonica*) [15]; and European eel [7]. In the latter study, 22 silver eels were released on the west coast of Ireland, of which 14 successfully transmitted light, temperature and depth data. Due to unknown causes, all but one tag experienced premature pop-up. Although the experiment fell short of revealing the full migration to the Sargasso Sea, the transmitted data revealed diel vertical migrations between depths of 200 and 1000 meters over highly variable temperature ranges. Interestingly, 2 of the 14 transmitting eels were probably eaten by predators, given the total absence of light recorded by the tags over several days immediately prior to pop-up. The dominant hypothesis formulated to explain the evolution of diel vertical migrations of anguillid eels (as in most vertically migrating fish) is predator avoidance, although thermoregulation to control metabolic rate and gonad maturation may also play a role [7], [15], [16].

In an attempt to document the migratory pathway(s) and the environmental conditions encountered by American eels during their oceanic migration to the Sargasso Sea, we tagged 8 silver eels with miniature satellite pop-up tags during their migration from the St. Lawrence River in Québec, Canada. Eels from the St. Lawrence are almost entirely comprised of large females, measuring approximately 1 m or more in length and approximately 2.5 kg in mass. They are thus big enough to limit the negative effects of externally fixed satellite tags that inevitably contribute to an increase in drag [17], [18]. In addition, the St. Lawrence population segment has one of the longest marine migrations of all American eels, migrating approximately 1600 km from the upper St. Lawrence River through the Gulf of St. Lawrence (GSL) prior to reaching the North Atlantic Ocean and a further 2500 km before reaching the southern Sargasso Sea. All released tags suffered premature pop-up but surprisingly, six of the eight tagged eels were ingested by warm-gutted predators, as observed by a sudden increase in ‘ambient’ temperature several days prior to pop-up. Gut temperatures were in the range of 20 to 25°C, too cold for marine mammals (exceeding 38°C in grey seals (*Halichoerus grypus*) [19]), but within the range of endothermic fish [20], [21]. These unexpected predation events enabled us to identify a potentially important marine predator of American eels as well as understand the environmental conditions and behavior of the eels coinciding with the predation events. More specifically, we aimed to (1) identify the eel predator by comparing its vertical migratory behavior with that of potential warm-gutted predators occupying the Gulf of St. Lawrence, (2) describe the environmental conditions, particularly depth and time of day, at the moment of the predation event, (3) document the diel vertical migratory behavior of the eels before and just prior to predation to detect any changes in behavior that might be associated with the predation event.

## Materials and Methods

### Study area

**Figure 1:**
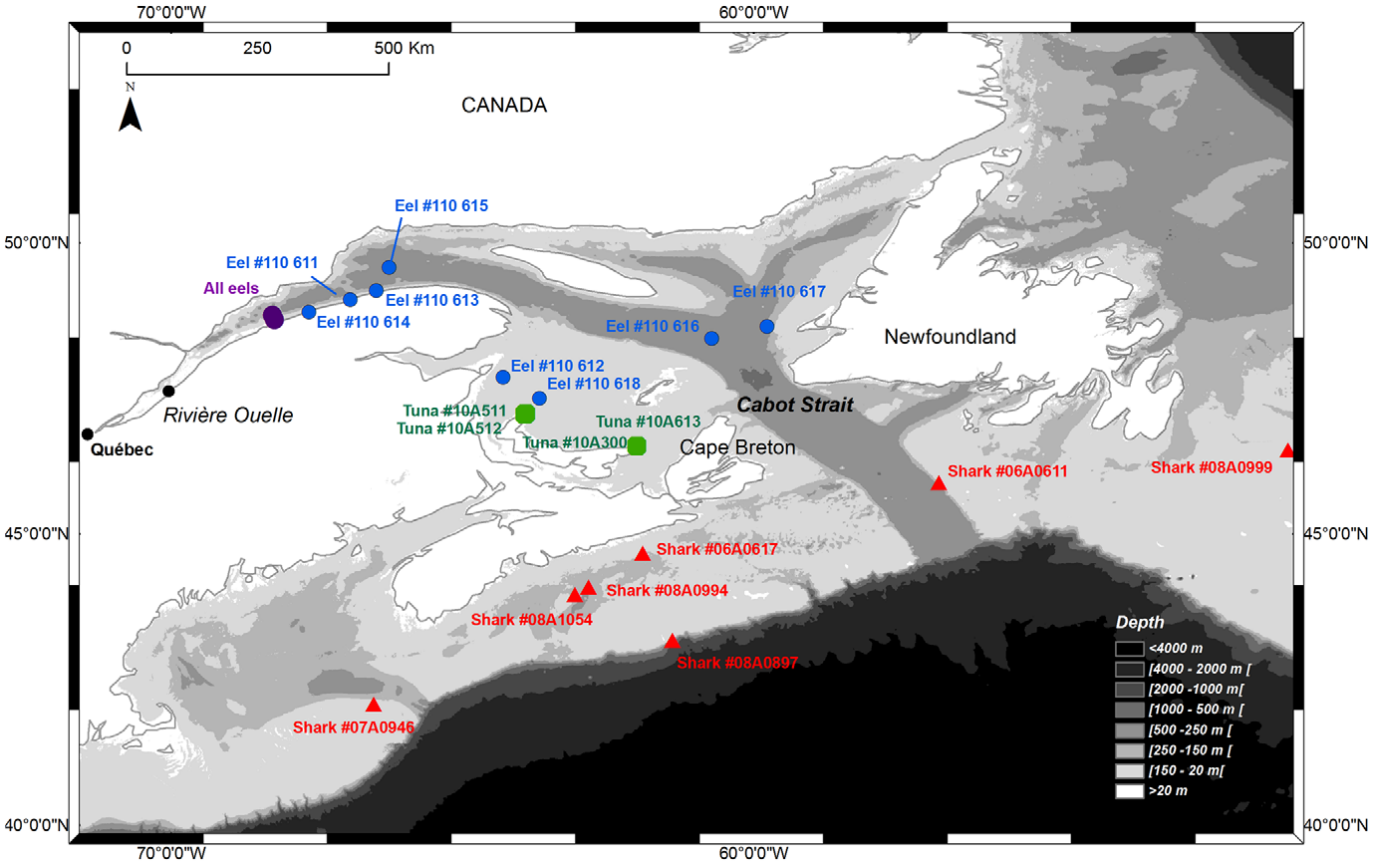
Map of the estuary and Gulf of St. Lawrence showing release site of eels (purple circles), sharks (red triangles) and tunas (green polygons) as well as location of first transmission of tagged eels (blue circles).

The tagging experiment was conducted in the lower estuary of the St. Lawrence River in October 2011 (Fig. 1). The lower estuary is a large body of water (12 600 km^2^) approximately 50 km across at the release point of the eels. The estuary empties into the Gulf of St. Lawrence (GSL), a semi-enclosed sea of 226 000 km^2^ [22]. The Gulf is bisected by the deep Laurentian Channel that reaches depths of between 300 and 500 m before opening onto the continental shelf via Cabot Strait located between Cape Breton Island and Newfoundland (Fig. 1). In summer and fall, the GSL has a cold intermediate layer sandwiched between warmer and fresher surface waters and warmer and saltier bottom waters from the Atlantic. The cold intermediate layer, with temperatures near 0°C, is a relic of winter cooling typically found in the Gulf between 30 and 100 m [23].

### Capture and eel tagging

During the first and second week of October 2011, large silver eels were captured alive in commercial trap nets during their downstream migration at Rivière Ouelle and transported in aerated holding tanks 191 km downstream for tag attachment and release in the lower estuary (Fig. 1). To minimize the negative effects of drag caused by the external tags, eight eels were selected for tagging on the basis of their large size and body mass (>97 cm and 2.5 kg; see Table 1). Each eel was equipped with an X-tag (pop-up satellite archival tag, PSAT, Microwave Telemetry, Columbia, Maryland, USA, http://www.microwavetelemetry.com) (Fig. S1). The transmitter measures 12 cm in length, has a maximum diameter of 32 mm, has an 18.5-cm aerial and weighs 45 g in air. The tags are slightly buoyant insuring their ascent to the surface following tag release. Onboard sensors collect and archive data on depth, water temperature and light every 2 min. The tags were programmed to record 12-bit resolution measurements of light, temperature (range −4°C to +40°C) and pressure (range 0 m to 1296 m) at 15-minute intervals and to store the records in the 64 Mb FLASH memory. At the end of each day (Coordinated Universal Time) the archived data for the previous 24 hours is processed within the tag to build up a subset of the data (the transmission buffer) for transmission to the Argos low earth orbiting satellite system (http://www.argos-system.org/) after tag release. The transmitters were pre-programmed to surface (‘pop-up’) on March 15, 2012 and, after surfacing, to transmit a subset of the archived data to Argos. In case of premature death of the host or detachment of the tag from its host, the transmitters were programmed to initiate the pop-up procedure and transmit data after seven consecutive days of constant depth readings (+/−3 m) with a 15 day delay following deployment (i.e., the tag ignores constant pressure for the first 15 days following deployment).

**Table 1:** Capture, tagging and release data for the 8 tagged eels and temperature and depth records of tags prior to and following predation.

				Release location (NAD 83)			First transmitting location (NAD 83)						Pre-predation Mean depth ± SD m				
Tag ID	Total length (mm)	Fresh Weight (kg)	Date of release	Lat (°N)	Long (°W)	First transmitting date	Lat (°N)	Long (°W)	Proportion of transmitted data	Duration of pre-predation track (days)	Duration of post-predation track (days)	Number of days at surface before transmitting data	during the day	at night	Post-predation Mean depth ± SD m	Pre-predation Mean Temperature ± °C	Post-predation Mean Temperature ± °C
110 611	970	2.6	10/04/11	48.67	68.22	10/26/11	49.18	66.47	96%	6.5	not eaten	15.2	−2.9±−5.8	−1.7±−3.6	-	5.5±0.9	-
110 612	1050	2.5	10/11/11	48.75	68.25	11/01/11	47.69	64.30	100%	2.0	6.1	11.8	−32.7±−48.3	−16.0±−12.9	−58.7±−71.0	6.2±1.4	20.6±3.0
110 613	1040	2.5	10/04/11	48.67	68.22	11/24/11	49.02	66.92	1%	?	?	?	-	-	-	-	-
110 614	1030	2.9	10/11/11	48.75	68.25	11/05/11	48.81	67.63	88%	1.0	5.4	18.3	−8.3±−13.2	−5.8±−8.3	−65.4±−73.3	6.8±0.8	21.8±2.3
110 615	990	2.5	10/04/11	48.67	68.22	10/26/11	49.58	66.25	95%	4.5	3.0	14.5	−4.7±−6.2	−7.5±−7.6	−14.1±−9.8	6.2±1.0	23.6±2.6
110 616	1160	2.9	10/04/11	48.67	68.22	12/11/11	48.35	60.72	65%	52.0	6.5	9.9	−204.0±−91.8	−119.6±−99.1	−75.0±−84.1	5.2±1.6	20.9±3.5
110 617	1115	3.0	10/11/11	48.75	68.25	11/17/11	48.56	59.77	71%	21.0	7.9	8.0	−146.2±−73.7	−35.1±−37.2	−19.2±−19.3	5.1±1.8	23.1±1.6
110 618	1131	2.6	10/11/11	48.75	68.25	10/31/11	47.33	63.68	100%	1.5	6.7	12.1	−66.3±−50.3	−13.9±−17.8	−33.6±−56.5	8.9±7.4	24.2±2.8

Two attachment methods were used to fit transmitters to the eels. A total of four fish were tagged using each of the two methods. The first method was a slightly adapted version of the method developed by Manabe et al. [15]. 14 kg monofilament fishing line was threaded through the dorsal musculature at two points on either flank of the body, anterior to the dorsal insertion and above the lateral line. This was achieved using 19-gauge hypodermic needles to penetrate the skin at each of the four points, piercing upwards through the dorsal musculature and exiting at roughly the same spot on the back from which the tag tether would extend. As a result, the tag was positioned just above the dorsal side of the eel anterior to the dorsal fin. The ends of the monofilament at each of the four points were secured by compressing very small lead fishing weights around them and knotting the distal end of each line. The compressed lead weights were prevented from rubbing against the skin of the individual by placing a small aluminum washer and smooth rubber disk between the lead weight and the skin. During previous attachment tests, we noted that having small rubber discs cover each of the four entry points resulted in greatly reduced skin lesions caused by abrasion. This procedure required approximately 7 minutes to complete after which each eel took approximately 30 minutes to fully recover.

In the second procedure, hypodermic needles (20 gauge) were pushed through the dorsal musculature approximately 30 mm below the dorsal surface and 0.5 mm surgical steel wire was fed through the bore of the needles before they were removed. The ends of the wire were then threaded through a protective neoprene pad and a small plastic plate on both sides of the eel. A 10 cm length of 3 mm nylon braid was attached to each of the plastic and the free ends attached to the transmitter. This secured and held the transmitter, which then floated approximately 3 cm above the back of the eel. The attachment point was posterior to the head, half-way between the head and the start of the dorsal fin. The procedure was rapid and generally took less than 2 minutes to complete.

Eels were weighed to the nearest g and body length measured to the nearest cm. They were then placed in a 10 000 L aerated tank filled with 2 000 L of full-strength salinity sea water to recover. All fish were released on the south shore of the St. Lawrence lower estuary at the Institut Maurice Lamontagne, Department of Fisheries and Oceans, Canada, (Fig. 1) 18 hours after tagging; 4 were released on October 4, 2011 and 4 others on October 11, 2011. All eels were transported by boat 5–10 km offshore in coolers and released in 30 m of water the first week and in 200 m of water the second week (surface temperature and salinity of approximately 10°C and 30 at release site).

Nine weeks prior to the experiment, five silver eels were tagged with ‘dummy’ tags of the same dimensions and mass as the x-tags and retained in the same tank to assess the functionality/longevity of tag attachment method 1. Eel behavior was also monitored following tag attachment. No major injuries related to tag attachment were observed and all tags remained securely attached during the survey. Twelve weeks following the onset of the experiment, one eel was accidentally entangled while cleaning the basin and eventually died. Three eels died 16–24 weeks after the experiment began and one eel survived 32 weeks in captivity after which it was released.

This study was carried out in strict accordance with the recommendations of the Canadian Council on Animal Care. The protocol was approved by the Animal Care Committee, Laval University (Permit Number 2011101-01) and Maurice-Lamontagne Institute, Fisheries and Oceans Canada (Permit Number 11-2). All surgery were performed under acetyleugenol (120 ppm) and all efforts were made to minimize suffering. Capture and transport of eels were authorized by the Ministère des Ressources Naturelles et de la Faune Québec (Permit Number 20110719-361-03GP).

### Identification of the unknown eel predators – Statistical analysis of vertical profiles

The unknown predators were necessarily warm-gutted fishes, given the depth and temperature profiles recorded by the ingested tags. This excludes seals and whales because the predators never came to the surface to breathe and the internal temperature was too low for mammal guts (e.g. [19]). Furthermore, the predator is likely relatively large given the large size of tagged eels. In the GSL, there are only two predatory species that fit these criteria: the Atlantic bluefin tuna (*Thunnus thynnus*) and the porbeagle shark (*Lamna nasus*). In order to identify the unknown eel predators, we developed a novel statistical procedure to compare the diel vertical migration behavior of tagged eels following predation with that of satellite-tagged Atlantic bluefin tuna and porbeagle sharks (PAT MK10's from Wildlife computer, www.wildlifecomputers.com). Four bluefin tuna were tagged and released in the GSL in the vicinity of Prince Edward Island (Fig. 1) providing data for September and October, 2010 at 10-second intervals (see [24] for methodology and procedures) (Table 2). Two tunas were captured by commercial fishermen in the GSL before the pop-up date. A total of seven porbeagle sharks were tagged and released on the Scotian Shelf in summer or autumn 2007, 2008, 2009 and 2010 (see [25] for methodology and procedures). One shark was recaptured and two tags were found after the pop-up date, allowing for recovery of the full data set, i.e. at 30-second intervals. For the four other sharks, the summarized interval between data points was 6 hours.

**Table 2:** Capture, tagging, release data and depth-profile variables for each of the 4 bluefin tunas and 7 porbeagle sharks used to compare with the 6 unknown eel predators.

						Release location (NAD 83)			Pop-up location (NAD 83)			Period						
Species	#ID	Sex	Total length (mm)	Fresh Weight (kg)	Date of release	Lat (°N)	Long (°W)	Pop-up date	Lat (°N)	Long (°W)	Delay between raw data	From	To	Maximal Depth (m)	Mean Depth (m)	Proportion of time spent within the 10 first m	Ecart Depth (Night/Day) %	Number of dives per day
Bluefin Tuna	10A300			341	08/31/10	47.06	−63.91	11/20/10	23.66	−73.15	30 sec	09/09/10	09/26/10	−35.7	−10.7	50.7	0.04	17.4
												09/26/10	10/14/10	−105.2	−10.5	59.2	0.14	23.5
	10A511		293	395	08/28/10	47.06	−63.91	10/05/10	47.10	−63.88	30 sec	09/01/10	09/18/10	−21.1	−4.2	91.1	0.04	22.6
												09/18/10	10/06/10	−35.9	−7.2	76.8	0.07	22.7
	10A512		262	290	08/29/10	47.05	−63.92	10/05/10	47.12	−60.92	30 sec	09/01/10	09/18/10	−32.7	−6.5	80.5	0.11	21.6
												09/18/10	10/05/10	−168.9	−16.9	54.7	0.33	20.6
	10A613			409	09/09/10	46.50	−62.00	11/25/10	23.77	−81.01	30 sec	09/10/10	09/25/10	−40.5	−12.2	45.5	0.13	20.5
												09/25/10	10/18/10	−85.8	−8.6	64.7	0.2	21.3
Porbeagle Shark	08A0999	M	131		08/10/10	46.44	−50.83	10/15/10	43.61	−59.40	10 sec	08/12/10	08/27/10	−33.1	−23.2	2.3	0.18	6.3
												08/27/10	09/14/10	−42.4	−25.4	2.1	0.07	6.1
												09/14/10	10/04/10	−211.5	−42.3	2.6	0.32	11.9
												10/04/10	10/25/10	−202.8	−40.6	5	0.28	12.3
	08A1054	F	101		11/03/10	44.09	−62.83	05/24/11	41.09	−62.83	10 sec	11/03/10	11/18/10	−119	−47.6	5.8	0.25	10.3
												11/18/10	12/03/10	−101.7	−50.9	0.8	0.39	8.3
	07A0946	F	217		07/06/08	42.08	−66.52	04/15/09	28.31	−74.86	10 sec	07/07/08	11/04/08	−248.2	−74.5	22.3	0.08	9.2
	R67735	M	181		09/28/06	45.88	−56.82	01/19/07	38.24	−54.76	6 hours	10/12/06	12/20/06	−246	−73.8	0	0.43	NA
	R70158	M	191		10/01/06	44.67	−61.90	02/02/07	42.65	−69.22	6 hours	10/03/06	12/26/06	−189	−47.3	0	0.38	NA
	R75374A	F	131		09/26/07	43.96	−63.07	02/13/08	42.29	−59.14	6 hours	09/27/07	11/13/07	−105	−23.1	3.8	0.38	NA
	R44404	F	191		06/12/09	43.17	−61.39	10/02/09	44.39	−55.84	2 to 6 hours	06/23/09	09/27/09	−407.5	−77.4	10.8	0.71	NA
Unknown Predator	110 612										15 min	10/04/11	10/13/11	−193.7	−58.1	30.2	0.03	9.4
	110 614										15 min	10/11/11	10/12/11	−188.3	−56.5	13.9	0.05	7.2
	110 615										15 min	10/04/11	10/08/11	−118.3	−11.8	19.3	0.14	23.3
	110 616										15 min	10/04/11	11/25/11	−242.1	−72.6	35.8	0.24	9.0
	110 617										15 min	10/11/11	11/01/11	−209.8	−42.0	46.9	0.06	11.8
	110 618										15 min	10/11/11	10/12/11	−83.4	−16.7	17.4	0.16	15.2

The vertical profiles (depth data) of the four tunas and seven sharks were then compared, using two methods. To our knowledge, this is the first time that a statistical identification has been attempted for unknown PSAT profiles. For both methods, the vertical profiles of individual sharks and tunas were sub-sampled to produce discreet time series (Table 2). Individual discreet time series were considered independent since each series comprised different behaviors observed over different periods of time and associated with different locations and environmental conditions. For the first method, the correlations between real depth profiles of unknown predators and those of sharks and tuna were determined (Spearman rank correlation). Correlations were calculated for all possible periods corresponding to the duration of the pattern observed for the unknown predator. For example, for predator #100614, the observed period was 5 days. Thus, all 5-day periods for each known predator were compared. A mean correlation coefficient per individual was then obtained. Only full days were considered such that day x started at midnight and ended on day x +1 at midnight. Data at 15 min intervals were computed (average from 30-sec and 10-sec intervals, for sharks and tunas respectively). Since data for four sharks were only available at 6-hour intervals, these sharks were not included in this method. Both 15-min interval data and 3-hour moving averages were used to assess the correlations. For graphical representation, the relative depths were computed by dividing the real depth by the maximum depth recorded during the reporting period.

The second method made use of a linear discriminant analysis based on several variables extracted from the vertical profiles. The purpose of linear discriminant analysis is to find the linear combination of the individual variables that will give the greatest separation between the groups of known and unknown predators. This method maximizes the ratio of between-class variance to the within-class variance in any particular data set thereby guaranteeing a maximum degree of separation [26]. A total of 27 variables characterizing the vertical profiles were calculated for each individual but most of these variables were correlated. Ultimately, four variables with the highest discriminatory power were retained: the mean depth (m), the proportion of time spent within the first 10 m of the water column, the difference of amplitude in depth between night and day (difference between max and min depth between night and day) and the average number of dives per day. The average number of dives per day is the average number of movements performed per day by fish, where one movement is defined as a descent followed by an ascent (or *vice versa*) greater than 10% of the observed depth variation. The homogeneity of the variance in each group was determined using a Bartlett's test.

## Results

### Evidence of predation

**Figure 2:**
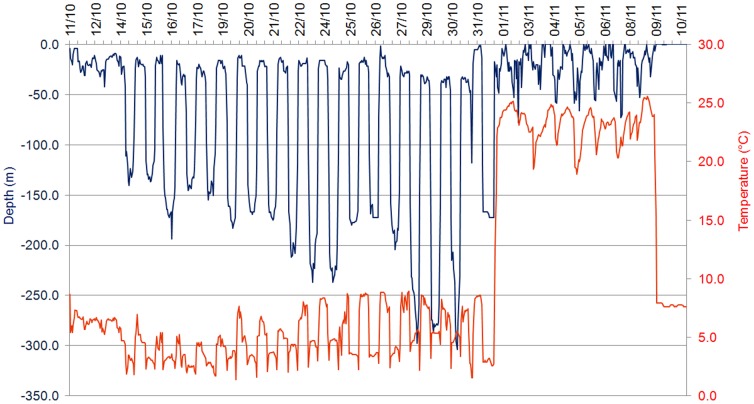
Vertical profile (depth & temperature) of a tagged eel (#110 617) illustrating the predation event and subsequent change in recorded temperature and depth (data represent one hour mean).

**Figure 3:**
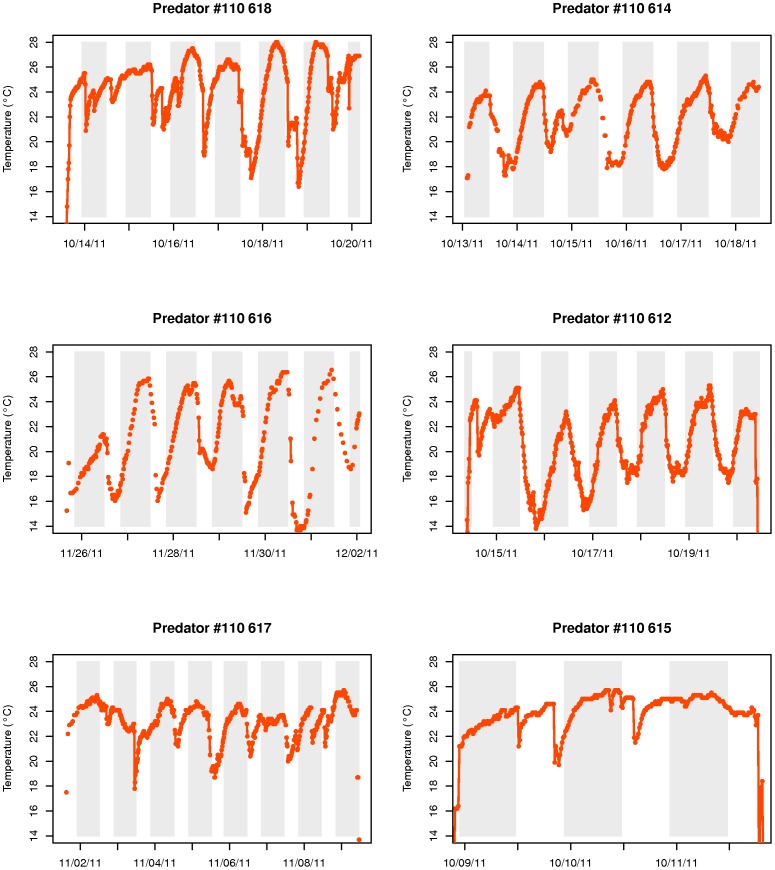
Temperature profile inside the guts of the 6 eel predators. The gray parts represent night period.

All eight satellite tags detached and surfaced prematurely (Table 1). One tag transmitted only 1% of its stored data and we were unable to reconstitute its history. The remaining seven tags transmitted between 65 and 100% of archived data following pop-up (Table 1). One of these tags revealed an eel track lasting 6.5 days prior to detachment with no evidence of predation. This eel may have died after 6.5 days or the tag detached on its own for some unknown reason. The remaining six eels were ingested by warm-gutted predators, as indicated by a sudden increase in the ambient temperature recorded by the tags and changes in diel vertical migration behavior (Table 1, Fig. 2). Predation occurred between 1 and 52 days following release (Table 1). Prior to predation, ambient water temperature fluctuated between 0.7 and 9.0°C (Fig. 2). Following predation, ambient temperatures fluctuated between approximately 22 and 28°C and varied as a function of depth (Table 1, Fig. 3). Five predators exhibited maximum gut temperatures prior to sunrise and minimum gut temperatures during daylight hours (Fig. 3). This diel periodicity was less evident for the remaining eel predator (Fig. 3). Tags remained in the predator's guts for between 3 and 8 days before being expelled and floating to the surface to begin transmitting.

### Eel behavior prior to predation

During the first days following tagging, all eels remained in surface waters and did not exhibit DVM. Three of the eels that were preyed upon were ingested at this time (Table 3). Another eel illustrated an inversed DVM and was preyed upon only 4.5 days after tagging. Eel #110 117, although exhibiting normal DVM throughout most of its track, inversed its DVM 24 hours prior to predation (Table 3, Fig. 2). Four eels were preyed upon during daytime, whereas the two night- predation events occurred at full-moon (Table 3).

**Table 3:** Environmental variables and eel behavior observed just prior to and at the moment of predation.

# X-tag	Day of predation	Moment/Period of predation	Fraction of the moon's visible disc (from new moon 0.00 to full moon 1.00)	Depth of predation	Duration of eel activity between release and predation (days)	Eel behaviour just before predation
110 612	14-Oct-11	Night- 3 hours before sunrise	0.97	<20 m	2	Eel in surface waters during the entire track
110 614	13-Oct-11	Night - 2 hours after sunset	0.99	≈−22 m	1	Eel in surface waters during the entire track
110 615	8-Oct-11	Day – 1 h20 before sunset	0.85	≈−15 m	4.5	Inversed DVM (in surface waters during the day)
110 616	25-Nov-11	Day- 4 hours after sunrise	0.00	Between −50 m and −26 m	52	Decreasing DVM at depth prior to predation
110 617	1-Nov-11	Day- 2 hours after sunrise	0.33	≈−20 m	21	Inversed DVM 24 hours prior to predation
110 618	13-Oct-11	Day - 2 hours after sunrise	0.99	≈−20 m	1.5	In surface waters prior to predation

### Identification of predators

**Figure 4:**
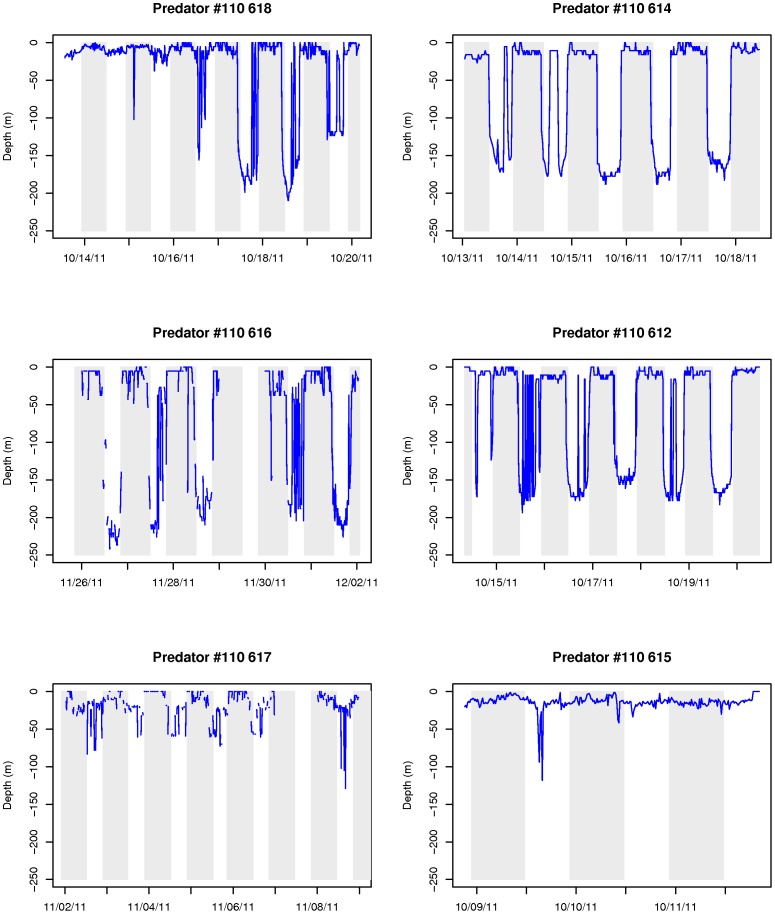
Vertical migratory behavior of the six eel predators. The gray parts represent night period.

The six eel predators exhibited diel vertical migrations after having ingested the eels, but patterns appeared to differ somewhat from those of eels, with some predators diving repeatedly from surface waters to depth during the day (Fig. 4). The vertical migrations of one of the predators were less well defined with it spending most of the time in surface waters (Fig. 4).

**Figure 5:**
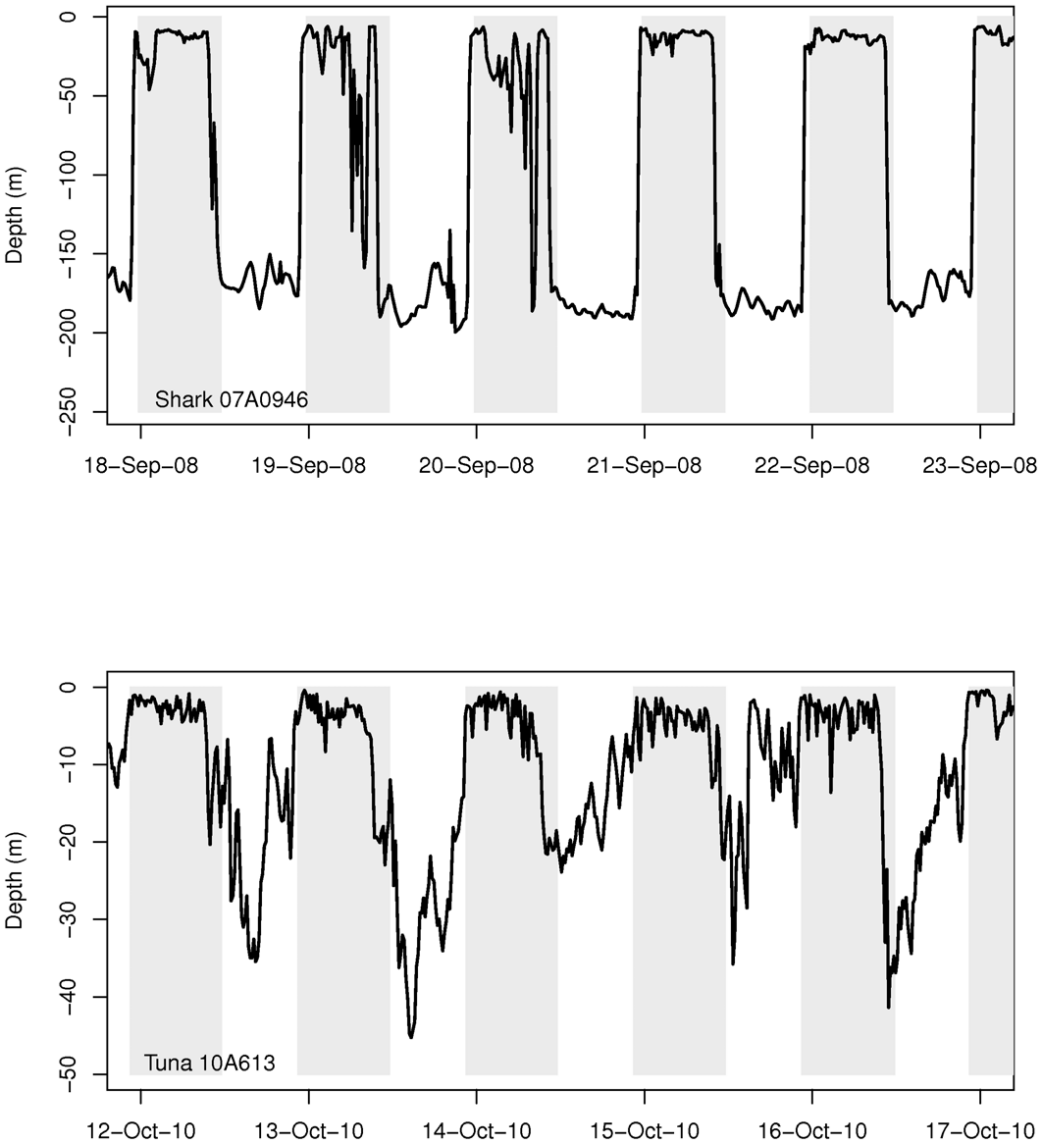
Actual vertical migration profiles of known predators. Upper panel (a): a porbeagle shark vertical profile (#07A0946) and lower panel (b): a bluefin tuna vertical profile (#10A613). The gray parts represent night period.

A visual comparison of the vertical migrations of the unknown predators with those of tuna and porbeagle shark suggested that the shark was the most likely candidate (Fig. 4 & Fig. 5). The shark profiles show a clear diel vertical pattern, occupying deeper waters during daytime and surface waters at night. This was far less clear for most tuna and their depth profiles were more erratic (Fig. 5). Their ascents to the surface occurred more gradually than those of sharks (Fig. 5). Indeed, this impression was supported by the two statistical methods used to identify the eel predators. The proportion of non-significant values for the correlation between known predators and unknown eel predators' depth profiles was higher for tuna (16.2%) than for sharks (9.5%) (See Table S1). Also, for five unknown eel predators, the highest significant correlation coefficients between vertical profiles were obtained with a shark (0.81<r^2^<0.91, p<0.001).

The linear discriminant analysis successfully discriminated tuna from sharks with the four selected variables. On average, sharks are found deeper than tuna (−43 m vs −9.1 m respectively, Table 2). Sharks also spend less than 5.8% of their time within the first 10 m water of the water column whereas tuna spend 65.4% of their time within this layer. The difference in amplitude (max depth - min depth) between day and night is more important for sharks (average difference of 16.9% against 6.1% in tunas). According to the 15-minute intervals considered in our analysis, tuna were also shown to make more dives per day (21.3±1.8 vs 9.2±2.5 for sharks). When applying the LDA to the six unknown eel predators in order to predict their class, all but one were classified as sharks (probability of 0.75 to 1.00). The exception is predator # 110 615 which is more likely a tuna according to this analysis. Indeed its vertical profile does not resemble that of the others (Fig. 4), although the period of tracking is only 2.5 days. Results of the correlation analysis were also ambiguous for this predator (Table S1).

## Discussion

At least six of seven eels tagged with X-tags were ingested by homeothermic fish that our study identified as most likely being porbeagle sharks. The sudden increase in temperature from 3–9°C to 20–25°C leaves little doubt as to predation by homeotherms, given that water temperatures in the Gulf of St. Lawrence during October are invariably below 9°C. As gut temperatures are well below that expected of marine mammals (exceeding 38°C in grey seals (*Halichoerus grypus*) [19]), we are left with two homeothermic fishes that occur in the Gulf as likely candidates: the Atlantic bluefin tuna and the porbeagle shark. Carey and Teal [27] reported that porbeagle muscle tissue was maintained 7–13°C above that of the surrounding water, even at water temperatures of 6°C. Goldman et al [21] reported an even larger difference between gut temperature (25–25.7°C) and the surrounding water temperature (5–16°C) in salmon sharks (*Lamna ditropis*), a close relative of the porbeagle shark. This finding is consistent with the difference observed between our ingested tags and ambient temperatures. In addition, we were able to accurately distinguish between tuna and shark by using the behavioral criteria generated by comparing the diving behavior of these two species with those of our unknown predators. Tuna spent comparatively more time near the surface than sharks and exhibited more vertical activity than sharks (more dives per day). Furthermore, there was less night/day variation in dive behavior for tunas when compared with sharks. Our statistical analysis showed that the profile characteristics of most eel predators more closely resembled those of sharks than those of tuna. Extremely rapid and extensive vertical dives (“bounce dives”, [28]) also seem to be characteristic of porbeagle sharks and the eel predators, although this is more apparent during the day for the eel predator and the night for porbeagle shark. This behavior however was never observed for bluefin tuna.

Porbeagle sharks are known eel predators, with American eels having been reported in their stomachs [29]. Furthermore, anecdotal observations of the gut contents of sharks caught in the Gulf of St. Lawrence between 2001 and 2008 revealed that approximately one in ten sharks contained silver-stage American eels in their stomachs (Cairns, D. DFO, Charlottetown, Canada, *pers. comm.*). Two other cases of shark predation on migrating silver eels fitted with X-tags were recently reported in Japan [15] and in the North Sea [30]. In both cases, an increase of ‘ambient’ temperature revealed predation by homeotherms. On the other hand, there is little evidence that tuna prey on eels. No eels were found in the stomachs of 568 bluefin tuna caught on the continental shelf of New England from July through October over a period of five years [31]. Furthermore, there was no evidence of American eels in the guts of 68 bluefin tuna sampled in the Atlantic off Cape Breton in 2010 [32]. Nevertheless, we cannot completely exclude the possibility that tuna could also be potential predators of migrating silver eels. The number of data used to compare profiles was small and the period and location were not identical for all individuals compared. Despite clear discrimination in the mean values of the selected characteristics between sharks and tuna, individual variability in behavior has the potential to be quite important. While patterns for five of the unknown predators (#612, 614, 616, 617, 618) appear to be very similar and, according to both analyses, correspond quite clearly to the patterns observed for sharks, the interpretation of patterns for one unknown predator (#615) remains ambiguous.

The temperature cycling observed within the guts of eel predators are most probably a combination of changing ambient water temperatures and feeding. Gut temperatures were warmest during the night when predators were in warmer surface waters (ambient *T* circa 8°C) and coolest during the day when predators were in colder, deeper waters (ambient *T* circa 3°C). However, in both tuna and lamnid sharks, fluctuations in gut temperatures are principally caused by the ingestion of cold-blooded prey and seawater followed by digestion and gut warming [20], [21]. In southern bluefin tuna, feeding events are characterized by increases in gut temperatures from 20 to 26°C followed by gradual cooling with periodicities varying from once to several times daily [20]. In lamnid sharks, the periodicity of similar temperature fluctuations is highly variable [21], [28]. The periodicity in gut temperatures documented here may be interpreted as more or less continuous ingestion occurring through much of the daylight hours with digestion and associated gut warming occurring principally at night.

The majority of tagged eels in this study appear to have fallen victim to predation, raising the possibility that predation during the spawning migration represents a non-negligible source of mortality. Recently, a very high predation rate was reported for 74 silver eels equipped with X-tags and released in Europe (Westerberg, H., pers. comm., Swedish Board of Fisheries, PO Box 423, S-40126 Göteborg, Sweden). A parallel study on St. Lawrence eels conducted during the same period revealed that only four of 113 migrating silver eels internally tagged with acoustic transmitters in the St. Lawrence estuary were detected by a listening line of moored hydrophones covering the entire Cabot Strait, at the mouth of the GSL (Fig. 1) (Béguer et al. in prep.). Although these analyses are still at a preliminary stage, the low detection rate observed may be in part the result of high predation rates. Given that only about 150 000 eels are estimated to migrate out of the St. Lawrence River annually (Verreault G, Ministère des Ressources Naturelles et de la Faune, pers. comm.), these observations raise the possibility that escapement of St. Lawrence eels from the Gulf is critically low, in part due to predation.

We cannot discount the possibility that the satellite tag itself contributed to increasing the eels' susceptibility to predation. Indeed, this interpretation is supported by the observation that three eels were eaten within two days of tagging. Although marked diel vertical migrations appear to be common among anguillid eels during the marine portion of their spawning migrations (*A. anguilla* [7], [30], [33], *A. japonica* [15] and *A. dieffenbachii* [14], [16], [34]), we observed reversed diel vertical migration for two eels the day prior to predation. This also could be interpreted as a tag effect leading to abnormal behavior that increased susceptibility to predation. Eels fitted with external tags may become more vulnerable to predation, either as a result of bleeding that may occur immediately following tagging or an increase in visibility to predators as a result of the presence of the tag itself [15]. There are several examples of predation on fishes equipped with external tags in the literature [35]: shark predation on white marlin [36] and on opah [37]. In addition, some studies on European eels have revealed that the drag of the external tag (PSAT) significantly impairs their swimming performance [17], [18]. Eels equipped with external tags have higher oxygen consumption during swimming and present an irregular swimming. Nevertheless, it is noteworthy that these studies addressing tag effect were conducted on much smaller eels than those tagged in the present study (by 12.7% and 38.3% in body mass and 56.4 and 72.1% in body length, respectively).

Our findings, although preliminary, raise the possibility that eels represent a reliable, predictable food resource for porbeagle sharks. The congeneric salmon shark (*Lamna nasus*) that inhabits the subarctic and temperate waters of the North Pacific is also known to regularly feed on migrating anadromous salmonids. Indeed, salmon sharks appear to aggregate during the summer months at specific locations along migration routes and in bays near the spawning grounds of returning adult Pacific salmon [38], [39]. During this time, the stomach contents of sampled sharks at these locations revealed that adult salmon were indeed the main prey [38]. Earlier work by Nagasawa [40] provided evidence that salmon sharks were also following and feeding on congregations of Pacific salmonids in the open ocean. Interestingly, a spatial linkage between migrating eels and porbeagle sharks was provided by Campana et al [25] who found that all mature porbeagle females fitted with satellite tags on the Scotian shelf headed south towards the subtropical waters of the Sargasso Sea during the winter, where the pupping ground is believed to occur, coincident with the spawning migration of American eels. Nevertheless, it is important to stress that any such association between porbeagle sharks and American eels is, at present, purely speculative. Further tagging efforts and rigorous analyses of the diet of predators are needed to test the predation hypothesis as well as to elucidate the routes and timing of migration. Finally, the development of a new generation of smaller satellite tags will surely help reduce their impact on tagged eels.

## Supporting Information

Figure S1 A wild eel (circa 1 meter long) equipped with an X-tag and released on October 2011, near Mont-Joli, in the St. Lawrence estuary.
(TIFF)

Table S1 Results of the statistical comparison between predator vertical profiles: Spearman rank correlation mean (Mean cc value) and maximum values (Max cc value), and proportion of non-significant values (NS).
(DOCX)
