# Supplementary figures and images for: Shark Predation on Migrating Adult American Eels (Anguilla rostrata) in the Gulf of St. Lawrence
Source: PLoS One. 2012 Oct 17;7(10):e46830. doi: 10.1371/journal.pone.0046830 (PMC3474790; doi:10.1371/journal.pone.0046830)

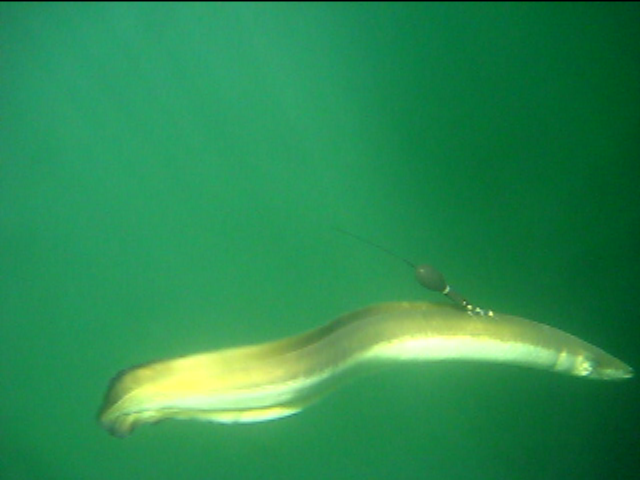

Supplement: Figure S1 — A wild eel (circa 1 meter long) equipped with an X-tag and released on October 2011, near Mont-Joli, in the St. Lawrence estuary. (TIFF) [file pone.0046830.s001.tiff]
