# Supplementary material for: Shark Predation on Migrating Adult American Eels (Anguilla rostrata) in the Gulf of St. Lawrence
Source: PLoS One. 2012 Oct 17;7(10):e46830. doi: 10.1371/journal.pone.0046830 (PMC3474790; doi:10.1371/journal.pone.0046830)
Supplement: Table S1 — Results of the statistical comparison between predator vertical profiles: Spearman rank correlation mean (Mean cc value) and maximum values (Max cc value), and proportion of non-significant values (NS). (DOCX) [file pone.0046830.s002.docx]

**Supporting Information Table**

|  |  | **Period** | | **Unknown predator 612** | | | **Unknown predator 614** | | | **Unknown predator 615** | | | **Unknown predator 616** | | | **Unknown predator 617** | | | **Unknown predator 618** | | |
| --- | --- | --- | --- | --- | --- | --- | --- | --- | --- | --- | --- | --- | --- | --- | --- | --- | --- | --- | --- | --- | --- |
| **Species** | **#ID** | **Min** | **Max** | **Prop NS** | **Mean cc value** | **Max cc value** | **Prop NS** | **Mean cc value** | **Max cc value** | **Prop NS** | **Mean cc value** | **Max cc value** | **Prop NS** | **Mean cc value** | **Max cc value** | **Prop NS** | **Mean cc value** | **Max cc value** | **Prop NS** | **Mean cc value** | **Max cc value** |
| Bluefin Tuna | 10A300 | 9-Sep-10 | 26-Sep-10 | 0.00 | 0.60 | 0.75 | 0.08 | 0.59 | 0.72 | 0.20 | 0.48 | 0.76 | 0.00 | 0.56 | 0.72 | 0.00 | 0.57 | 0.78 | 0.00 | 0.58 | 0.72 |
|  | 10A300 | 26-Sep-10 | 14-Oct-10 | 0.00 | 0.58 | 0.70 | 0.00 | 0.58 | 0.73 | 0.19 | 0.40 | 0.64 | 0.00 | 0.53 | 0.68 | 0.00 | 0.60 | 0.76 | 0.00 | 0.53 | 0.66 |
|  | 10A511 | 1-Sep-10 | 18-Sep-10 | 0.18 | 0.19 | 0.35 | 0.50 | 0.28 | 0.46 | 0.36 | 0.02 | 0.49 | 0.40 | 0.22 | 0.38 | 0.42 | 0.27 | 0.40 | 0.10 | 0.17 | 0.40 |
|  | 10A511 | 18-Sep-10 | 6-Oct-10 | 0.42 | 0.24 | 0.56 | 0.38 | 0.24 | 0.73 | 0.40 | 0.20 | 0.55 | 0.36 | 0.15 | 0.35 | 0.23 | 0.17 | 0.52 | 0.36 | 0.22 | 0.55 |
|  | 10A512 | 1-Sep-10 | 18-Sep-10 | 0.17 | 0.10 | 0.42 | 0.31 | 0.12 | 0.48 | 0.47 | -0.11 | 0.49 | 0.18 | 0.12 | 0.36 | 0.15 | 0.08 | 0.44 | 0.27 | 0.11 | 0.40 |
|  | 10A512 | 18-Sep-10 | 5-Oct-10 | 0.15 | 0.28 | 0.60 | 0.14 | 0.30 | 0.80 | 0.31 | 0.24 | 0.62 | 0.00 | 0.23 | 0.58 | 0.15 | 0.30 | 0.72 | 0.08 | 0.28 | 0.69 |
|  | 10A613 | 10-Sep-10 | 25-Sep-10 | 0.00 | 0.71 | 0.79 | 0.00 | 0.65 | 0.82 | 0.00 | 0.51 | 0.64 | 0.00 | 0.65 | 0.73 | 0.00 | 0.64 | 0.82 | 0.00 | 0.67 | 0.74 |
|  | 10A613 | 25-Sep-10 | 18-Oct-10 | 0.06 | 0.52 | 0.82 | 0.16 | 0.60 | 0.82 | 0.14 | 0.33 | 0.71 | 0.18 | 0.53 | 0.74 | 0.11 | 0.58 | 0.86 | 0.18 | 0.57 | 0.80 |
| Porbeagle Shark | 08A0999 | 12-Aug-10 | 27-Aug-10 | 0.00 | 0.65 | 0.77 | 0.00 | 0.63 | 0.80 | 0.00 | 0.48 | 0.64 | 0.00 | 0.57 | 0.68 | 0.00 | 0.61 | 0.74 | 0.00 | 0.63 | 0.78 |
|  | 08A0999 | 27-Aug-10 | 14-Sep-10 | 0.00 | 0.68 | 0.79 | 0.00 | 0.70 | 0.82 | 0.06 | 0.46 | 0.68 | 0.00 | 0.63 | 0.68 | 0.00 | 0.70 | 0.81 | 0.00 | 0.64 | 0.75 |
|  | 08A0999 | 14-Sep-10 | 4-Oct-10 | 0.00 | 0.51 | 0.76 | 0.00 | 0.50 | 0.81 | 0.24 | 0.43 | 0.68 | 0.00 | 0.43 | 0.59 | 0.00 | 0.51 | 0.74 | 0.08 | 0.49 | 0.62 |
|  | 08A0999 | 4-Oct-10 | 25-Oct-10 | 0.00 | 0.62 | 0.77 | 0.06 | 0.60 | 0.78 | 0.11 | 0.44 | 0.65 | 0.00 | 0.59 | 0.73 | 0.00 | 0.58 | 0.80 | 0.00 | 0.60 | 0.75 |
|  | 08A1054 | 3-Nov-10 | 18-Nov-10 | 0.00 | 0.10 | 0.68 | 0.18 | 0.20 | 0.72 | 0.15 | 0.14 | 0.80 | 0.22 | 0.30 | 0.65 | 0.09 | 0.08 | 0.66 | 0.11 | 0.21 | 0.61 |
|  | 08A1054 | 18-Nov-10 | 3-Dec-10 | 0.60 | -0.30 | -0.17 | 0.27 | -0.20 | 0.34 | 0.38 | -0.17 | 0.27 | 0.44 | -0.19 | 0.09 | 0.55 | -0.26 | -0.11 | 0.44 | -0.13 | 0.10 |
|  | 07A0946 | 7-Jul-08 | 4-Nov-08 | 0.00 | 0.76 | 0.91 | 0.00 | 0.73 | 0.89 | 0.00 | 0.69 | 0.84 | 0.00 | 0.69 | 0.81 | 0.00 | 0.69 | 0.84 | 0.00 | 0.67 | 0.79 |

Table S1. Results of the statistical comparison between predator vertical profiles: Spearman rank correlation mean (Mean cc value) and maximum values (Max cc value), and proportion of non-significant values (Prop NS).
